# Synthesis, Characterization and Application of a New Functionalized Polymeric Sorbent Based on Alkenylphoshine Oxide

**DOI:** 10.3390/polym15061591

**Published:** 2023-03-22

**Authors:** Sławomir Frynas, Monika Wawrzkiewicz

**Affiliations:** 1 Department of Organic Chemistry and Crystallochemistry, Faculty of Chemistry, Maria Curie-Skłodowska University in Lublin, 33 Gliniana Str., 20-614 Lublin, Poland; 2 Department of Inorganic Chemistry, Faculty of Chemistry, Maria Curie-Sklodowska University in Lublin, M. Curie-Sklodowska Sq. 2, 20-031 Lublin, Poland

**Keywords:** α,β-unsaturated phosphine oxide, phosphorus-containing polymers, polymeric sorbents, basic dye adsorption, Basic Blue 3, Basic Yellow 2

## Abstract

A novel phosphorus-containing sorbent (CyP(Ph)4–DVB) was prepared by copolymerizing divinylbenzene (DVB) with bis α,β-unsaturated phosphorylated cyclohexene (CyP(Ph)4). ATR-FT-IR indicated that the phosphinoyl group was introduced into the sorbent structure. The thermal properties of the sorbent were investigated using a differential scanning calorimeter (DSC), which revealed that (CyP(Ph)4–DVB) is more stable than poly(DVB). The CyP(Ph)4–DVB was applied for cationic dye removal, such as C.I. Basic Yellow 2 (BY2) and C.I. Basic Blue 3 (BB3). Batch adsorption tests suggested that the Freundlich isotherm model seemed to be the better one for the description of equilibrium sorption data at equilibrium, rather than the Langmuir or Temkin models. The Freundlich constants concerning the adsorption capacity of CyP(Ph)4–DVB, *k_F_*, were calculated as 14.2 mg^1−1/n^L^1/n^/g for BY2 and 53.7 mg^1−1/n^L^1/n^/g for BB3.

## 1. Introduction

Functional synthetic polymers are increasingly prominent materials due to their unique properties and applications [1,2,3]. The specific application fields of functional synthetic polymers are determined by the nature of the functional groups and their locations and the structure of the polymer matrix [4]. One of the groups of functionalized polymers comprises polymers possessing a phosphoric function. The phosphorus-containing polymers have been successfully applied in biotechnology and biomedical engineering, nanotechnology, the food industry, hydrometallurgy, catalysis and the purification of various industrial and wastewaters, and as polymer supports, adsorbents and ion exchange resins [5,6,7,8,9]. 

Some phosphorus polymers possess a phosphorus function in the main chain (polyphosphazenes [10], polyphosphoesters [11]). Most common are polymers containing a phosphorus moiety in the side part of the main chain (phosphinic acid derivatives [12], phosphonate derivatives [13], phosphorus-containing fluoropolymers [14]). Materials with special topologies or architectures, such as stars, hyperbranched polymers or dendrimers (treelike structures), are also of great interest [15].

Functional synthetic polymers can be prepared either via the chemical modification of already defined polymers [16] or via the direct polymerization or copolymerization of functionalized monomers [17,18,19,20]. The first method has the drawback of lacking control over the molecular weight and functional group distribution, while the second requires careful adaptation of the polymerization technique to avoid side reactions of the desired functional groups. The copolymerization process allows the easy and direct introduction of the functional group into the polymer structure. The functional group exists in all volumes of the polymer structure.

Polymers with a phosphine oxide function are readily synthesized via free radical copolymerization with common monomers, e.g., styrene or methyl methacrylate. The resulting copolymers can be used as potential flame-retardant materials [21,22]. Phosphorus-containing polymers also are used as sorbents [8,22,23,24,25,26,27,28]. Resins are particularly useful in the removal, preconcentration and determination of various metal ions in aqueous solutions and in metal recovery [23]. A silica-based octyl(phenyl)-*N*,*N*-diisobutylcarbamoyl-methylphoshine oxide (CMPO) resin (CMPO/SiO_2_-P) was applied for Nd(III) removal from a nitric acid medium [24]. Modification of silica by the impregnation and immobilization of two chelating agents, such as *N*,*N*,*N*,*N*-tetraoctyl-3-oxapentane-1,5-diamide (TODGA) and octyl(phenyl)-*N*,*N*-diisobutylcarbamoylmethylphoshine oxide (CMPO), made it possible to obtain chelating polymeric resins for actinide and lanthanide (Am(III), Cm(III), Gd(III), Sm(III)) preconcentration [25]. A phosphine-functionalized PVA/SiO_2_ composite nanofiber adsorbent of a porous structure was evaluated for manganese and nickel ion removal from an aqueous solution at various pH values by Islam et al. [26]. Li et al. [27] synthesized a new type of phosphine-based covalent organic material (P-COFs) for iodine adsorption. A phosphine-functionalized magnetic nanocomposite (Fe_3_O_4_/SiO_2_/OPPh_2_) was used as a palladium ion adsorbent [28]. A phosphorus-containing polymer sorbent obtained by the chemical modification (oxidative chlorophosphorylation reaction) of an industrial polymer—butadiene rubber—was also applied for the removal of the toxic azo dye arsenazo III by Alosmanov [29]. The chloromethylated styrene-divinylbenzene copolymers modified with iso-propylamine and diethylphosphite were tested for phenol and its derivatives’ uptake from water [30].

Dyes are applied in several industries, such as the textile, plastic, paper, leather, food, pharmaceutical, cosmetic or rubber industries [31]. These industrial sectors are among the world’s greatest polluters. Wastewater in the textile industry is characterized by relatively high levels of biochemical oxygen demand (BOD) and chemical oxygen demand (COD). It is noteworthy that they contain large amounts of non-biodegradable organic compounds, especially textile dyes [32]. Around 2–3% of basic dyes do not bind to the fabric during the dyeing operation and are released into wastewater, which is commonly used, in developing countries, for the purpose of irrigation in agriculture [32,33,34]. In addition, dyes have been proven to exhibit mutagenic, carcinogenic and toxic properties to biota, and they can enter the human food chain and cause dysfunction in many organs [32,35,36,37]. The high toxicity of basic dyes such as C.I. Basic Violet 1 (LD_50_ = 0.05 mg/L, LD_50—_median lethal dose) and C.I. Basic Yellow 37 (LD_50_ = 0.8 mg/L) was confirmed [37]. The use of appropriate yet comprehensive methods to remove dyes from wastewater is therefore a huge challenge. Adsorption takes a leading place among other physical methods of wastewater treatment [38]. It has many advantages over other techniques because it requires less energy and is flexible and easy to use with versatile designs. In addition, the adsorption method of wastewater treatment was estimated to be cost-effective. Its costs are in the range of $5.0–200 US $/m^3^, while the costs of other technologies are in the range of $10.0–450 US $/m^3^ [38].

**Figure 1:**
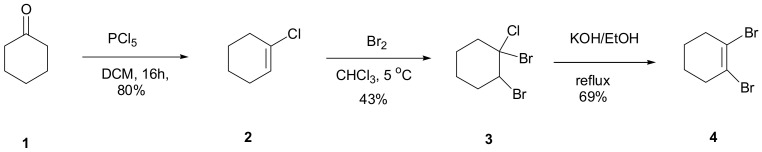
Synthesis of 1,2-dibromocyclohexene.

**Figure 2:**
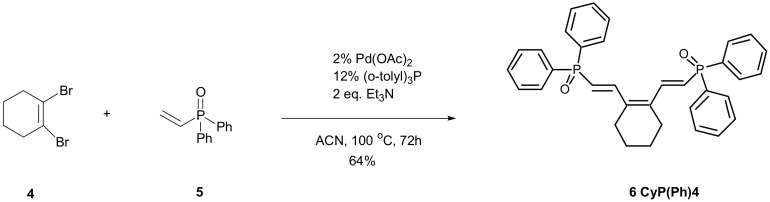
Synthesis of CyPPh4 **6**.

In this work, we attempted to use a phosphorus monomer containing two phosphine oxide moieties in copolymerization with styrene. Monomer **6** was prepared in four steps, including the synthesis of dibromocyclohexene (Figure 1) followed by the Heck reaction of dibromide with diphenylvinylphosphine oxide **5** (Figure 2). 

1-Chlorocyclohexene **2** was prepared in 80% yield using a procedure described by Axenov and co-workers [39]. Reaction **2** with bromine and the subsequent elimination of chlorine atoms using KOH in ethanol allowed us to obtain 1,2-dibromocyclohexene **4**. 

With the necessary dibromide **4** in hand, we were able to complete our synthesis of **6** with 64% yield in the Heck reaction, using diphenylvinylphosphine oxide **5** and tris o-tollyl phosphine as a ligand [40]. The CyP(Ph)4-DVB obtained as a result of the crosslinking reaction of 1,2-bis((*E*)-2-diphenylphospinoylethenyl)-cyclohex-1-ene with divinylbenzene was then applied as a potential polymeric sorbent for basic dye removal from aqueous solutions. A review of the literature allows us to conclude that polymeric adsorbents based on diphenylvinylphosphine oxide **5** have not been widely used so far to remove dyes from wastewater. Therefore, the adsorption capacities of CyP(Ph)4-DVB in comparison to poly(DVB) towards cationic dyes such as BY2 and BB3 were determined. 

## 2. Materials and Methods

### 2.1. Materials

All chemicals and solvents mentioned above were used as received, without further purification. Cyclohexanone, PCl_5_, bromine, KOH, benzyl alcohol and ethanol were from Avantor Performance Materials Poland S.A. (Gliwice, Poland). Palladium acetate, trimethylamine, tris(o-tollyl)phosphine and bis(2-ethylhexyl)sulfosuccinate sodium salt were purchased from Sigma-Aldrich (Germany). Divinylbenzene (DVB) and α,α′-azoiso-bis-butyronitrile (AIBN) were obtained from Merck (Germany) and Fluka (Switzerland), respectively.

**Figure 3:**
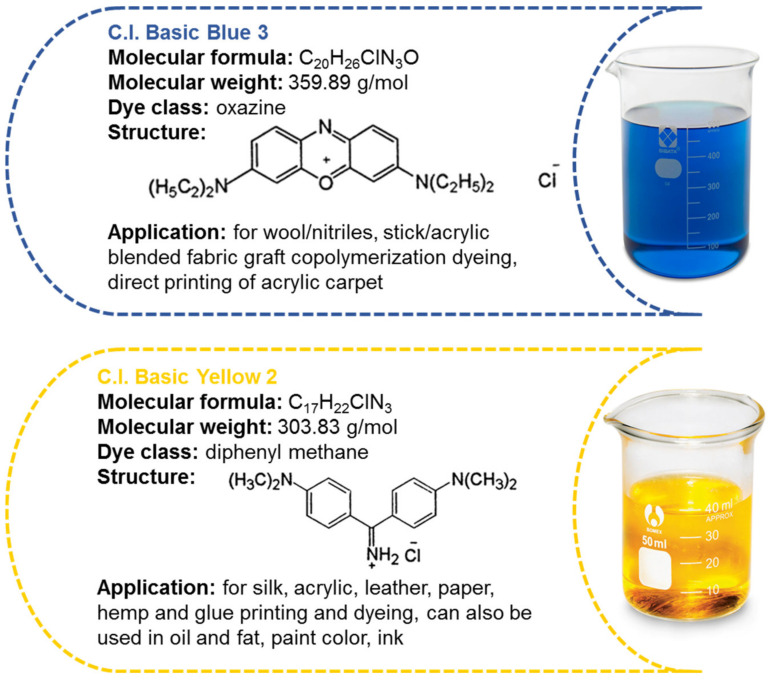
Basic dyes’ characteristics.

Two basic dyes of the oxazine and diphenylmethane class, namely C.I. Basic Blue 3 (7-(diethylamino)-*N*,*N*-diethyl-3H-phenoxazin-3-iminium chloride) and C.I. Basic Yellow 2 (4,4′-carbonimidoylbis(*N*,*N*-dimethylaniline) hydrochloride), were used as adsorbates. The structure and characteristics of the dyes are presented in Figure 3.

### 2.2. Synthesis of CyP(Ph)4

Nuclear magnetic resonance (NMR) spectra were recorded on the Bruker AV300 (1H 300 MHz, ^31^P 121.5 MHz, ^13^C NMR 75 MHz) and Bruker AV500 (^1^H 500 MHz, ^31^P 202 MHz, ^13^C NMR 126 MHz) spectrometers (Bruker; Billerica, MA, USA). All spectra were obtained in CDCl_3_ solutions unless mentioned otherwise, and the chemical shifts (δ) are expressed in ppm using the internal reference to TMS and external reference to 85% H_3_PO_4_ in D_2_O for ^31^P. Coupling constants (*J*) are expressed in Hz. The abbreviations of signal patterns are as follows: s—singlet, d—doublet, t—triplet, q—quartet, m—multiplet. Elemental analyses were performed on the PerkinElmer CHN 2400.

- 1-chlorocyclohexene 2

The product was synthesized according to the modified literature procedure [39]. Dry CH_2_Cl_2_ (30 mL) was added under the flow of argon into a Schlenk flask charged with PCl_5_ (20.0 g, 96.0 mmol). The resulting suspension was cooled in a water bath and vigorously stirred, and then cyclohexanone (9.4 g, 96.0 mmol) was slowly added via a syringe. The reaction setup was refluxed in an argon atmosphere overnight. The resulting brownish solution was quenched by addition to ice (100 mL). After extraction of the water layer with diethyl ether (3 × 20 mL), the combined organic phases were neutralized by the addition of K_2_CO_3_ until no CO_2_ evolution was observed. The neutralized organic extracts were dried over K_2_CO_3_ and evaporated in vacuo. The residual brownish oil was distilled under reduced pressure to give the product in a clean state as a colorless oil (b.p. 141–145). Yield: 9.0 g, 80%. The physical and spectral data for 1-chlorocyclohexene 2 are in accordance with those previously reported [39].

b. 1,2-dibromocyclohexene 4

A mixture of 20 g (0.172 mol) 1-chloro-cyclohexene 2 in 20 mL of CHCl_3_ was placed in a flask fitted with a dropping funnel and reflux condenser. To the mixture was added 9 mL (0.35 mol) of bromine in 10 mL of dry CH_3_Cl, keeping the temperature at 5–10 °C using an ice bath. Next, the solution was stirred at 5 °C for 2 h. The mixture was allowed to warm slowly to room temperature. The solution was next washed with saturated Na_2_SO_3_ and the organic layer was dried with MgSO_4_ and evaporated in vacuo. The residual oil was distilled under reduced pressure (b.p. 120–128 °C, 12 mm Hg) to give the product in a clean state as an oil, which crystalized after cooling in the ice bath to give 20.5 g of crude product **3** (yield 43%). 

Crude 1,2-dibromo-1-chloro-cyclohexane **3** (20 g, 0.072 mol) was placed in a flask fitted with a dropping funnel and reflux condenser. Next, 100 mL of 20% solution of KOH in ethanol was added to the reaction. The mixture was heated under reflux for 2–3 h (the reaction was monitored using the TLC technique). After completing the reaction, the product was separated by means of steam distillation, resulting in an oil crystallizing on cooling. Product **4** was recrystallized from ethanol. Yield: 12 g (69%).

1,2-dibromocyclohexene 4: Colorless solid, mp 42–43 °C, NMR ^1^H NMR (CDCl_3,_ 500 MHz): *δ* 1.76–1.78 (m, 4H), 2.56–2.60 (m, 4H). 

^13^C NMR (CDCl_3_, 126 MHz): *δ* 24.0, 37.3, 122.9. Anal. Calcd. for C_6_H_8_Br_2_ C, 30.03; H, 3.36; Found C, 30.08; H, 3.29.

c. 1,2-bis ((E)-2-diphenylphospinoylethenyl)-cyclohex-1-ene 6

A mixture of 4.65 g (0.0194 mol) of 1,2-dibromocyclohexene 4, 8.67 g (0.0379 mol) of diphenylvinylphosphine oxide 5, 7.38 g (0.0729 mol) of triethylamine, 0.18 g (0.8 mmol) of palladium acetate and 1.04 g (3.2 mmol) of tris *o*-tollylphosphine in 10 mL of acetonitrile was heated in a capped heavy-walled Pyrex tube flushed with argon in a steam bath at 110 °C for 72 h. After cooling, the mixture was washed with three small portions of a 5% solution of hydrochloric acid and dried over MgSO_4_. Next, the solvent was evaporated and the product was isolated with a silica gel column (CHCl_3_:acetone 8:1) to give 6.62 g (64%) of 1,2-bis ((E)-2-diphenylphospinoylethenyl)-cyclohex-1-ene as a yellow pale crystal.

Yellow pale solid, mp 226–227 °C, Anal. Calcd. for C_34_H_32_O_2_P_2_ C, 76.39; H, 6.03; Found C, 76.16; H, 6.06. 

NMR ^31^PNMR (CDCl_3,_ 202 MHz): *δ* 25.17 ppm.

^1^H NMR (CDCl_3,_ 500 MHz): *δ* 1.69 (s, 4H), 2.39 (s, 4H), 6.38 (dd, *J* = 17.0 and 20.8 Hz, 2H), 7.44–7.49 (m, 8H), 7.51–7.54 (m, 4H), 7.57 (dd, *J* = 17.3 and 19.8 Hz, 2H), 7.67–7.71 (m, 8H).

^13^C NMR (CDCl_3_, 126 MHz): *δ* 21.8, 26.7, 120.9 (d, *J* = 103.5 Hz), 128.6 (d, *J* = 12.7 Hz), 131.4 (d, *J* = 10.0 Hz), 131.8 (d, *J* = 2.7 Hz), 132.7 (d, *J* = 105.4 Hz), 137.4 (d, *J* = 18.2 Hz), 143.4 (d, *J* = 5.5 Hz).

### 2.3. Synthesis of Polymeric Sorbent

In the first stage, the appropriate amount of phosphorous compound (CyP(Ph)4 6 was dissolved in benzyl alcohol for 2 h (1 g/5 mL). The bis(2-ethylhexyl)sulfosuccinate sodium salt (0.75 g, surfactant) and purified water (75 mL) were placed in a 250 mL three-necked flask equipped with a mechanical stirrer, an air condenser and a thermometer. The mixture was stirred intensively to dissolve the surfactant for 15 min at 80 °C. Next, the monomer DVB was added to the previously prepared mixture CyP(Ph)4 6 with benzyl alcohol. The initiator (AIBN) was added in the amount of 2 wt.% of monomers. The monomers were added in two molar proportions, 1:0.25 and 1:0.5 (DVB to CyP(Ph)4 6). Additionally, for comparison purposes, the homopolymer DVB was obtained. The whole solution was mixed and added to the aqueous phase. The reaction mixture was stirred at 350 rpm for 6 h at 80–85°C. The obtained polymer microspheres were filtered off and washed with distilled hot water (2 L) and purified with acetone [41,42,43].

### 2.4. Characteristics of Polymeric Sorbent

The images of the polymeric sorbents were obtained using a Malvern optical microscope (Malvern, Great Britain). 

The attenuated total reflection (ATR) was recorded based on Fourier transform infrared spectroscopy (ATR-FT-IR) using a TENSOR 27 Bruker spectrometer equipped with a diamond crystal (Ettlingen, Germany). The spectra were recorded in the range of 4000–600 cm^−1^ with 32 scans per spectrum at a resolution of 4 cm^−1^.

Differential scanning calorimetry (DSC) curves were obtained with the use of a DSC Netzsch 204 calorimeter Netzsch (Günzbung, Germany). The measurements were taken in aluminum pans with pierced lids. The sample mass was approx. 10 mg under a nitrogen atmosphere (30 mL/min). Dynamic scans were performed at a heating rate of 10 °C/min in the temperature range of 0–200 °C. Additionally, to evaluate the T_g_ (glass transition temperature), the heating rate of 2 °C/min in the temperature range −20 to 100 °C was applied. An empty aluminum crucible was used as a reference.

The pH of zero point charge (pH_PZC_) of CyP(Ph)4-DVB was determined by applying the solid addition method [44]. The CyP(Ph)4-DVB in the amount of 0.2 g was immersed in 20 mL of 0.01 M KNO_3_ solutions in which the initial pH (pH_0_) values from 1.4 to 9.9 were adjusted using 1 M HCl or 1 M NaOH and left for 24 h. The final pH (pH_f_) of the solutions was measured after 24 h using pH-meter CP-411 (Elmetron, Zabrze, Poland). The pH_PZC_ value was determined based on the curve of pH_0_ versus ΔpH (difference between the initial pH and the final pH, ΔpH = pH_0_ − pH_f_).

### 2.5. Batch Adsorption Experiments

The batch adsorption method was used to determine the sorption capacities (*q_e_*) of CyP(Ph)4-DVB and poly(DVB) towards basic dyes such as Blue 3 and Yellow 2 using Equation (1):

(1) $$q_{e}=\frac{\left(C_{0}-C_{e}\right)}{m}\;\cdot V$$

where *C_0_* and *C_e_* (mg/L)—BY2 and BB3 concentrations in the solution before adsorption, and at equilibrium, respectively; *V* (L)—the volume of the dye solution; and *m* (g)—the mass of CyP(Ph)4-DVB or poly(DVB).

The sorbents in the amount of 0.02 g were weighed in the conical flasks, and 20 mL of each of the dye solutions of specified concentrations was poured. Dye solutions with concentrations ranging from 1 to 100 mg/L were prepared to evaluate the sorption capacity of the polymeric sorbent. The samples were agitated in a mechanical shaker, Elpin 358+ (Lubawa, Poland), at 180 rpm (amplitude 8) at room temperature for 24 h. After a predetermined shaking time, the solution was decanted and the dye content was measured using a Cary 60 Agilent spectrophotometer (Santa Clara, CA, USA) at 430 nm (for BY2) and at 654 nm (for BB3). The adsorption experiments were performed in triplicate with reproducibility ±5%.

## 3. Results

### 3.1. Visualization of Sorbent

**Figure 4:**
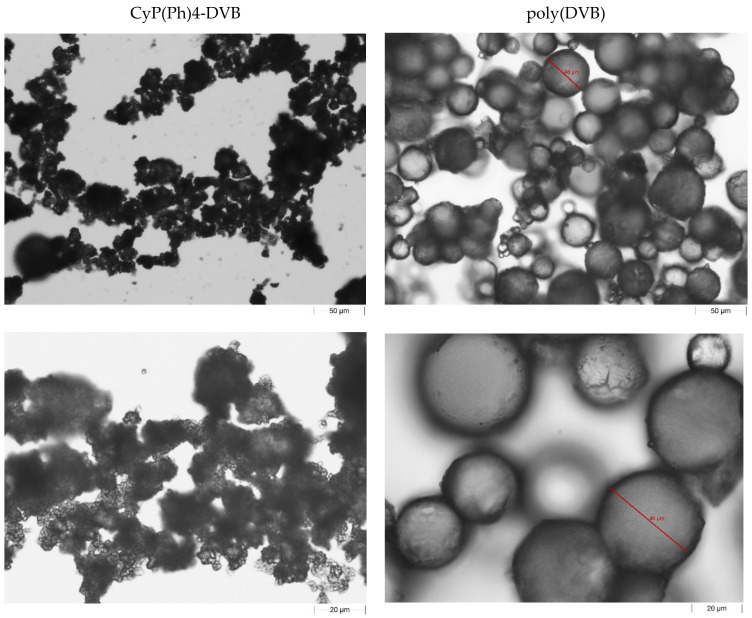
Optical images of obtained sorbent CyP(Ph)4-DVB.

The images of the synthesized polymeric sorbent and poly(DVB) obtained using an optical microscope are presented in Figure 4. As can be seen, the CyP(Ph)4-DVB sorbent is characterized by an irregular shape. The particle size ranges from 60 to 200 µm. The average particle size of the polymeric adsorbents based on DVB-co-GMA (GMA—glycidyl methacrylate) and DVB-co-GMA-TETA (TETA—triethylenetetramine) was found to be 117–120 µm [20]. For comparison, the bead size of the commercially available polystyrene-based adsorbent Amberlite XAD4 resin is 490–690 µm [45].

### 3.2. ATR-FT-IR Analysis

**Figure 5:**
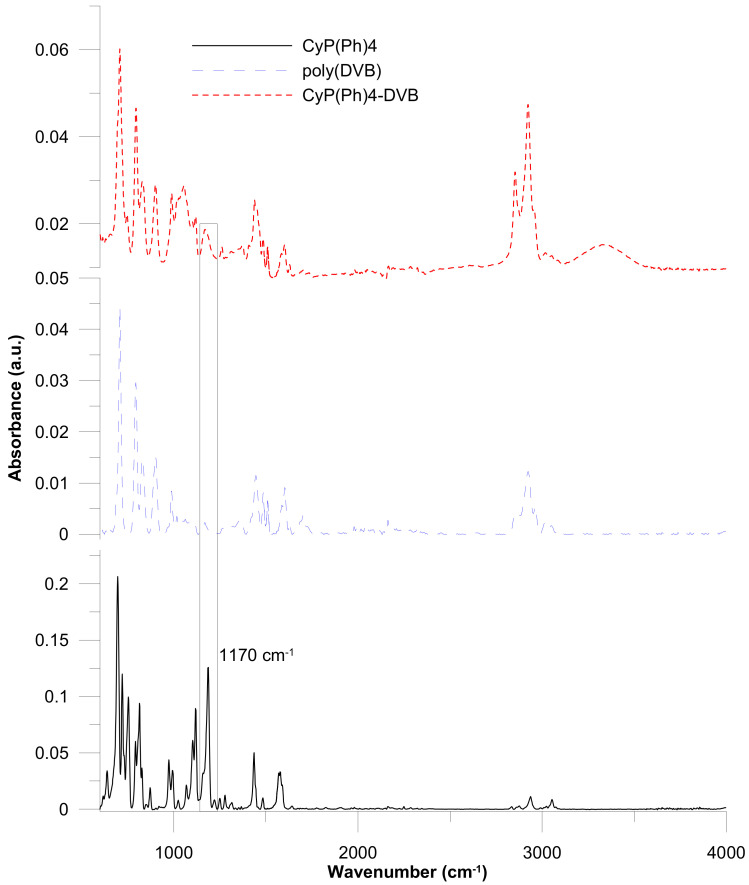
ATR-FT-IR spectra of substrate **6** and obtained polymeric sorbents.

The ATR-FT-IR spectra of the CyP(Ph)4 **6** are presented in Figure 5. The band in the range of 3053 cm^−1^ originates from the stretching vibration of the C-H in an alkene. On the other hand, in the 1578 cm^−1^ region, stretching vibrations of the C=C double bond were observed. The signal at 974 cm^−1^ indicates the presence of bending vibration of the C=C double bond. In the 1187 cm^−1^ region, there is a vibration of the P=O bond [46]. In the ATR-FT-IR spectra of the CyP(Ph)4-DVB sorbent, the signals from the C=C double (1578 cm^−1^) and C-H in alkene bond (3053 cm^−1^) disappeared. In the 2923–2858 cm^−1^ region, symmetric and asymmetric stretching vibrations of the methyl and methylene groups were observed. The vibration originating from the P=O can be observed in the range of 1170 cm^−1^.

**Figure 6:**
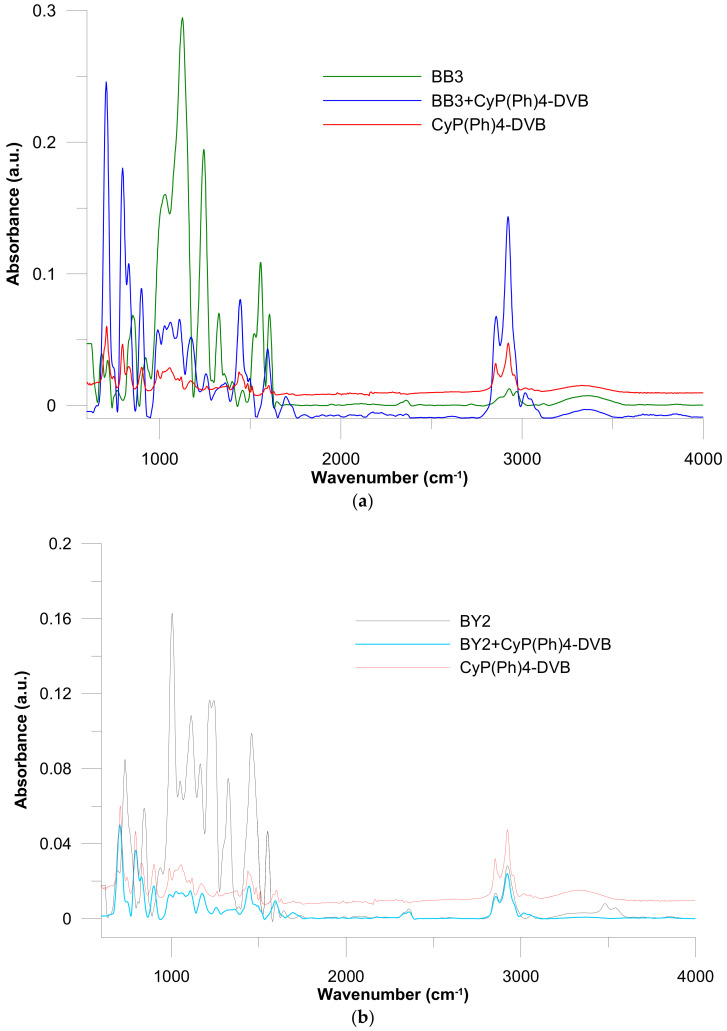
ATR-FT-IR spectra of dyes and polymeric sorbents before and after (**a**) BB3 and (**b**) BY2 adsorption.

Some peaks were shifted and new peaks were also detected in the ATR-FT-IR of the BB3 and BY2 adsorbed in CyP(Ph)4-DVB (Figure 6). In ATR-FT-IR, pure BB3 and BY2 signals from iminium salt at 2358 cm^−1^ were observed. After the sorption of BB3 and BY2, new peaks at 1694 cm^−1^ were detected. This signal indicates the sorption of the tested dyes in iminium form. Shifted bands at the 1000–1200 and 1460 cm^−1^ regions indicate C–N stretching bonds. 

### 3.3. DSC Analysis

**Figure 7:**
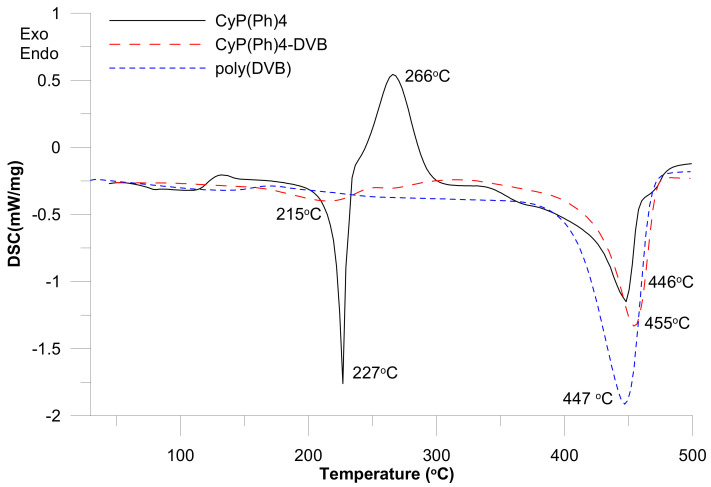
DSC curves of substrate **6** and obtained polymeric sorbents.

The DSC method was used to determine the thermal properties of 1,2-bis ((*E*)-2-diphenylphospinoylethenyl)-cyclohex-1-ene **6** and the CyP(Ph)4-DVB sorbent (Figure 7). 

The DSC curve of CyP(Ph)4 shows two endothermic effects and one strong exothermic effect. The endothermic effect that occurs at 227 °C is associated with the melting point of CyP(Ph)4. The strong exothermic effect with a maximum at 266 °C probably comes from cyclization or cross-linked reactions. This type of thermolysis reaction was previously observed for unsubstituted triene compounds [47]. The decomposition temperatures are in the range of 400–460 °C with a peak maximum (T_max_) at 446 °C. The DSC curve for the CyP(Ph)4-DVB sorbent shows thermal decomposition in the range of 410–470 °C with a maximum at 455 °C [48]. In comparison to the poly-DVB sorbent (T_max_) at 447 °C, the new sorbent is more stable.

### 3.4. Determination of pH_PZC_ of CyP(Ph)4-DVB

**Figure 8:**
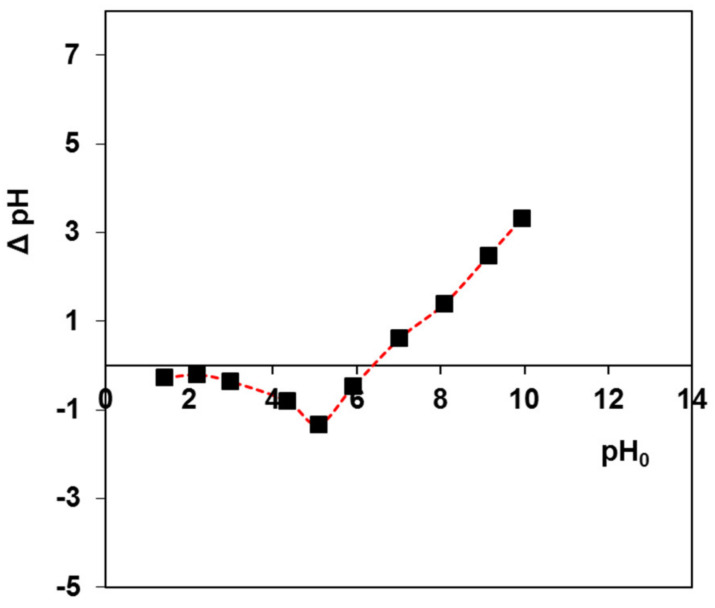
Determination of pH_PZC_ of the CyP(Ph)4 polymeric adsorbent.

The pH_PZC_ of the CyP(Ph)4 adsorbent is a very important factor in determining the pH value at which the adsorbent surface exhibits electrical neutrality. It was found that the determined pH_PZC_ value for CyP(Ph)4 is 6.4, as presented in Figure 8. At pH values lower than pH_PZC_, the adsorbent surface is positively charged, while, at pH values higher than pH_PZC_, the adsorbent surface is negatively charged. The negatively charged surface of CyP(Ph)4 interacts with the cationic forms of the BY2 and BB3 dyes. The pH_PZC_ of modified biochar/alginate composite bead (MCB/ALG) adsorbents applied for cationic dye methylene blue removal was in the range of 4.5–5.5 [49]. Removal studies of BY2 and BB3 using a carbon–silica (C/SiO_2_) composite revealed that the pH_PZC_ of C/SiO_2_ was 3.1 [50].

### 3.5. Evaluation of Adsorption Capacity of CyP(Ph)4-DVB

Determination of the sorption capacity (q_e_) by the batch method made it possible to assess the adsorption capacity of the synthesized CyP(Ph)4-DVB material and compare its properties with those of poly(DVB) and other adsorbents. For this purpose, three isotherm models, namely the Langmuir, Freundlich and Temkin models (Equations (2)–(4)) [51,52], were used:

(2) $$\frac{C_{e}}{q_{e}}=\frac{1}{Q_{0}k_{L}}+\frac{C_{e}}{Q_{0}}$$

(3) $$log\;q_{e}=log\;k_{F}+\frac{1}{n}log\;C_{e}$$

(4) $$q_{e}=\left(\frac{RT}{b_{T}}\right)lnA+\left(\frac{RT}{b_{T}}\right)lnC_{e}$$

where *C_e_*—the equilibrium concentration of the dye in the solution (mg/L); *Q_0_*—the monolayer adsorption capacity (mg/g); *k_L_*—the Langmuir constant (relating to the free energy of adsorption) (L/mg); *k_F_* (mg^1−1/n^ L^1/n^/g) and 1/*n*—the Freundlich constants concerning the adsorption capacity and the surface heterogeneity, respectively; *R*—the gas constant (8.314 J/mol K); *T*—the temperature (K); *A* (L/g) and *b_T_* (J/mol)—the Temkin constants.

Linear regression was applied to calculate the isotherm parameters from the *C_e_*/*q_e_* vs. *C_e_*, *log*(*q_e_*) vs. *log*(*C_e_*) and *q_e_* vs. *lnC_e_* plots. The fit of the isotherm equations to the experimental data was estimated taking into account the values of the determination coefficients R^2^. Table 1 summarizes the obtained results. 

The Langmuir isotherm model describes adsorption on a homogeneous adsorbent surface with the formation of the monolayer in which the adsorbate particles, i.e., dyes, do not interact with each other. The values of R^2^ were low and ranged from 0.438 to 0.880 for BY2 and BB3 adsorption on poly(DVB) and CyP(Ph)4-DVB, which rules out the Langmuir model to describe the experimental data. 

The Temkin isotherm model considers that the heat of adsorption of the chemical individuals in the layer decreases linearly as a result of increasing coverage of the adsorbent surface. The *b_T_* values considering adsorption energy and calculated for the adsorption of the basic dye on the CyP(Ph)4-DVB and poly(DVB) were positive, which indicated that the adsorption of the dyes on the obtained adsorbents was exothermic. However, the R^2^ values for the Temkin model, listed in Table 1, are lower than for the Freundlich model. 

**Figure 9:**
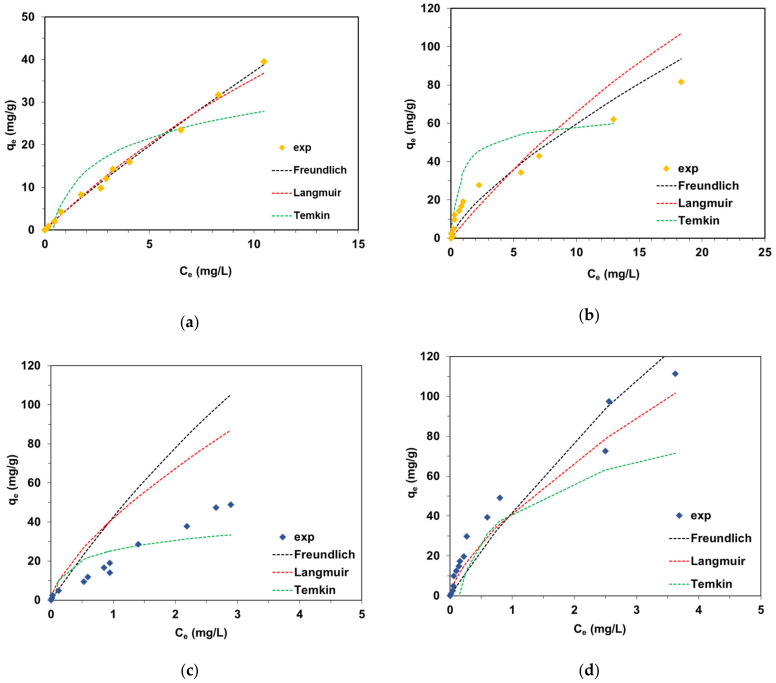
Fitting of the equilibrium experimental data to the Langmuir, Freundlich and Temkin isotherm models for the adsorption of (**a**) BY2 on poly(DVB), (**b**) BY2 on CyP(Ph)4-DVB, (**c**) BB3 on poly(DVB) and (**d**) BB3 on CyP(Ph)4-DVB.

The fitting results presented in Figure 9 indicate that the BY2 and BB3 adsorption process on poly(DVB) and CyP(Ph)4-DVB was a multilayer adsorption process, as the Freundlich model assumed. 

**Figure 10:**
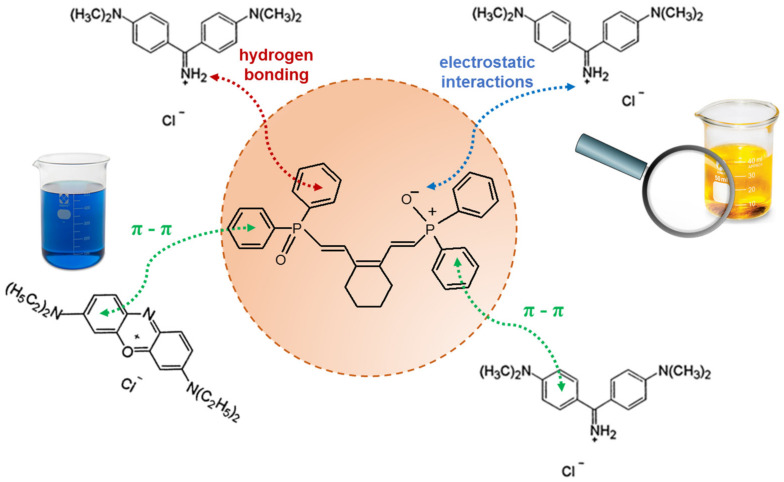
The proposed possible interactions between BY2 and BB3 dyes and CyP(Ph)4-DVB sorbent.

The R^2^ values of the Freundlich isotherm model were higher (0.995 and 0.816 for BY2 adsorption, 0.968 and 0.939 for BB3 adsorption) than those of the Langmuir isotherm model. Moreover, the 1/n values for BY2 and BB3 adsorption on the CyP(Ph)4-DVB were calculated to be 0.63 and 0.76, indicating that it could be considered a favorable adsorption system. The *k_F_* values were higher for BY2 (14.2 mg^1−1/n^L^1/n^/g) and BB3 (53.7 mg^1−1/n^L^1/n^/g) adsorption on the CyP(Ph)4-DVB than for the dyes’ (4.56 mg^1−1/n^L^1/n^/g for BY2 and 20.1 mg^1−1/n^L^1/n^/g for BB3) adsorption on poly(DVB), which underlines the validity of the synthesis. The adsorption mechanism of BY2 and BB3 on CyP(Ph)4-DVB mainly involves π–π interactions between the aromatic rings of the dyes and the benzene rings in the adsorbent [53,54,55]. As illustrated in Figure 10, the formation of weak hydrogen bonds, as well as electrostatic interactions, between the positively charged BY2 and BB3 dyes and the negatively charged surface of the CyP(Ph)4-DVB under the experimental conditions may also be considered as the binding mechanism [56]. It was previously confirmed that BY2 adsorption on a C-SiO_2_ composite [50], lignin-based hybrid adsorbents [53] and EGDMA-cellulose polymer sorbents [54] (where EGDMA—ethylene glycol dimethacrylate) took place in accordance with the Freundlich model and involved the above-proposed interactions. For example, the k_F_ values determined for C-SiO_2_ and lignin-based hybrids were equal to 27.37–55.41 mg^1−1/n^L^1/n^/g [50] and 11.1–45.7 mg^1−1/n^L^1/n^/g [53] depending on the temperature changes, respectively. BB3 adsorption on sulfuric-acid-activated montmorillonite [55], C-SiO_2_ [50] and functionalized lignin-based hybrids [53] also followed the Freundlich model. 

## 4. Conclusions

A new sorbent was obtained as a result of the crosslinking reaction of 1,2-bis ((*E*)-2-diphenylphospinoylethenyl)-cyclohex-1-ene with divinylbenzene and applied for the removal of the hazardous basic dyes such as C.I. Basic Yellow 2 and C.I. Basic Blue 3 from aqueous solutions. 

The ATR-FT-IR analysis confirmed the presence of the phosphinoyl group in the synthesized materials. The vibrations of the P=O bond (1187 cm^−1^) were visible in the spectra. After dye sorption in the ATR-FT-IR spectra, there were observed signals characteristic of iminium and tertiary amine.

DSC curves showed that the sorbent was thermally stable up to 400 °C and the CyP(Ph)4-DVB sorbent was more stable than only the DVB sorbent. The decomposition temperatures of the CyP(Ph)4-DVB sorbent were within the range of 400–460 °C, with a peak maximum at 455 °C, and this was 8 °C higher than the maximum peak from the DVB sorbent.

The applicability of the Freundlich isotherm model in the description of the equilibrium sorption data concerning BY2 and BB3 uptake by CyP(Ph)4-DVB and poly(DVB), rather than Langmuir and Temkin, was confirmed. Moreover, the *k_F_* values calculated for BY2 and BB3 adsorption on CyP(Ph)4-DVB were higher than for the dyes’ uptake by poly(DVB). This indicates an improvement in the sorption capacity of the new polymer sorbent CyP(Ph)4-DVB compared to poly(DVB).

## Figures and Tables

**Table 1:** Isotherm parameters determined for BY2 and BB3 adsorption on poly(DVB) and CyP(Ph)4-DVB.

Isotherm	Parameters	poly(DVB)	CyP(Ph)4-DVB	poly(DVB)	CyP(Ph)4-DVB
		BY2		BB3	
Langmuir	*Q*_0_ (mg/g)	141.7	93.1	78.3	136.7
	*k_L_* (L/mg)	0.03	0.19	0.42	0.77
	R^2^	0.549	0.653	0.438	0.880
Freundlich	*k_F_* (mg^1−1/n^L^1/n^/g)	4.56	14.2	20.1	53.7
	1/*n*	0.91	0.63	0.64	0.76
	R^2^	0.995	0.816	0.968	0.939
Temkin	*A* (L/mg)	2.6	8.1	31.8	26.1
	*b_T_* (J g/mol mg)	294.3	207.1	335.2	131.0
	R^2^	0.767	0.813	0.652	0.859

## Data Availability

The data supporting reported results can be received from the Authors.

## References

[1] Xia Q., Qin A., Tang B.Z. (2023). Recent advances in chiral AIE polymers. J. Nanopart. Res..

[2] Chyr G., DeSimone J.M. (2023). Review of high-performance sustainable polymers in additive manufacturing. Green Chem..

[3] Liu Y., Lu S., Luo J., Zhao Y., He J., Liu C., Chen Z., Yu X. (2023). Research progress of antistatic-reinforced polymer materials: A review. Polym. Adv. Technol..

[4] Makvandi P., Iftekhar S., Pizzetti F., Zarepour A., Zare E.N., Ashrafizadeh M., Agarwal T., Padil V.V.T., Mohammadinejad R., Sillanpaa M. (2020). Functionalization of polymers and nanomaterials for water treatment, food packaging, textile and biomedical applications: A review. Environ. Chem. Lett..

[5] Mane S. (2016). Functional polymers: A review. Can. Chem. Trans..

[6] Monge S., David G. (2014). Phosphorus-Based Polymers: From Synthesis to Applications.

[7] Hamed F., Biji P. (2015). A novel polymer containing phosphorus–nitrogen ligands for stabilization of palladium nanoparticles: An efficient and recyclable catalyst for Suzuki and Sonogashira reactions in neat water. Dalton Trans..

[8] Hiranphinyophata S., Iwasaki Y. (2021). Controlled biointerfaces with biomimetic phosphorus-containing polymers. Sci. Technol. Adv. Mater..

[9] Akhmedov V.M., Maharramov A.M., Azizov A.A., Alosmanov R.M., Bunyad-Zadeh I.A., Aliyeva S.B. (2019). Equilibrium, kinetic, and thermodynamic studies on the sorption of some heavy metal ions by the phosphorus-containing polymer sorbent. Russ. Chem. Bull. Int. Ed..

[10] Allcock H.R. (2006). A Perspective of polyphosphazene research. J. Inorg. Organomet. Polym. Mat..

[11] Chen D.-P., Wang J. (2006). Synthesis and characterization of block copolymer of polyphosphoester and poly(ε-caprolactone). Macromolecules.

[12] Kloda M., Ondrušová S., Lang K., Demel J. (2021). Phosphinic acids as building units in materials chemistry. Coordin. Chem. Rev..

[13] Chen L., Wang Y.-Z. (2010). Aryl polyphosphonates: Useful halogen-free flame retardants for polymers. Materials.

[14] Wehbi M., Mehdi A., Negrell C., David G., Alaaeddine A., Ameduri B. (2020). Phosphorus-containing fluoropolymers: State of the art and applications. ACS Appl. Mater. Interfaces.

[15] Caminade A.-M., Majoral J.-P. (2016). Bifunctional phosphorus dendrimers and their properties. Molecules.

[16] Alosmanov R., Wolski K., Matuschek G., Magerramov A., Azizov A., Zimmermann R., Aliyev E., Zapotoczny S. (2017). Effect of functional groups on the thermal degradation of phosphorus- and phosphorus/nitrogen-containing functional polymers. J. Therm. Anal. Calorim..

[17] Wang S., Wang J., Ji Q., Shultz A.R., Ward T.C., McGrath J.E. (2000). Miscibility and morphologies of poly(arylene ether phenyl phosphine oxide/sulfone) copolymer/vinyl ester resin mixtures and their cured networks. J. Polym. Sci. B.

[18] Podkościelna B., Bartnicki A., Gawdzik B. (2009). Porous microspheres, copolymers of bis [4-(2-hydroxy-3-methacryloyloxypropoxy)phenyl]sulfide, and divinylbenzene as stationary phase for HPLC. J. App. Polym. Sci..

[19] Podkościelna B., Kołodyńska D. (2013). A new type of cation-exchange polymeric microspheres with pendant methylenethiol groups. Polym. Adv. Technol..

[20] Wawrzkiewicz M., Podkościelna B., Podkościelny P. (2020). Application of functionalized DVB-*co*-GMA polymeric microspheres in the enhanced sorption process of hazardous dyes from dyeing baths. Molecules.

[21] Rabinowitz R., Marcus R., Pellon J. (1964). Synthesis, polymerization, and copolymerization of diphenyl-p-styrylphosphine, phosphine oxide, and phosphine sulfide. J. Polym. Sci. A.

[22] Ebdon J.R., Price D., Hunt B.J., Joseph P., Gao F., Milnes G.J., Cunliffe L.K. (2000). Flame retardance in some polystyrenes and poly(methyl methacrylate)s with covalently bound phosphorus-containing groups: Initial screening experiments and some laser pyrolysis mechanistic studies. Polym. Degrad. Stab..

[23] Trochimczuk A., Czerwińska S. (2005). In(III) and Ga(III) sorption by polymeric resins with substituted phenylphosphinic acid ligands. React. Funct. Polym..

[24] Zhang A., Wei Y., Kumagai M., Koma Y., Koyama T. (2005). Resistant behavior of a novel silica-based octyl(phenyl)-N,N-diisobutyl carbamoylmethylphoshine oxide neutral extraction resin against nitric acid, temperature and γ-radiation. Radiat. Phys. Chem..

[25] Zhang A., Kuraoka E., Hoshi H., Kumagai M. (2004). Synthesis of two novel macroporous silica-based impregnated polymeric composites and their application in highly active liquid waste partitioning by extraction chromatography. J. Chromatogr. A.

[26] Islam M.S., Rahaman M.S., Yeum J.H. (2015). Phosphine-functionalized electrospun poly(vinyl alcohol)/silica nanofibers as highly effective adsorbent for removal of aqueous manganese and nickel ions. Colloids Surf. A.

[27] Li Y., Li X., Li J., Liu W., Cheng G., Ke H. (2021). Phosphine-based covalent organic framework for highly efficient iodine capture. Micropor. Mesopor. Mater..

[28] Naini N., Sid Kalal H., Almasian M.R., Niknafs D., Taghiof M., Hoveidi H. (2022). Phosphine-functionalized Fe_3_O_4_/SiO_2_/composites as efficient magnetic nanoadsorbents for the removal of palladium ions from aqueous solution: Kinetic, thermodynamic and isotherm studies. Mater. Chem. Phys..

[29] Alosmanov R.M. (2016). Adsorption of arsenazo III dye by phosphorus-containing polymer sorbent. J. Serb. Chem. Soc..

[30] Davidescu C.-M., Ardelean R., Popa A. (2019). New polymeric adsorbent materials used for removal of phenolic derivatives from wastewaters. Pure Appl. Chem..

[31] Garba A., Tahir A., Yusuf A.K. (2021). Adsorption of methylene blue using activated carbon made from watermelon rinds. J. Sustain. Mater. Process. Manag..

[32] Lellis B., Fávaro-Polonio C.Z., Pamphile J.A., Polonio J.C. (2019). Effects of textile dyes on health and the environment and bioremediation potential of living organisms. Biotechnol. Res. Innov..

[33] Das J., Dangar T.K., Panigrahy M. (2022). Bioremediation of Heavy Metals: A substantive potential for clean earth. J. Sustain. Mater. Process. Manag..

[34] Hessel C., Allegre C., Maisseu M., Charbit F., Moulin P. (2007). Guidelines and legislation for dye house effluents. J. Environ. Manag..

[35] Robinson T., McMullan G., Marchant R., Nigam P. (2001). Remediation of dyes in textile effluent: A critical review on current treatment technologies with a proposed alternative. Bioresource Technol..

[36] Alderete B.L., da Silva J., Godoi R., Rabaioli da Silva F., Taffarel S.R., Pisoni da Silva L., Hilario Garcia A.L., Mitteregger Júnior H., Neubauer de Amorim H.L., Picada J.N. (2020). Evaluation of toxicity and mutagenicity of a synthetic effluent containing azo dye after advanced oxidation process treatment. Chemosphere.

[37] Holme I., Griffiths J. (1984). Ecological aspects of color chemistry. Developments in the Chemistry and Technology of Organic Dyes.

[38] Dotto G.L., Santos J.M.N., Rodrigues I.L., Rosa R., Pavan F.A., Lima E.C. (2015). Adsorption of Methylene Blue by ultrasonic surface modified chitin. J. Colloid Interface Sci..

[39] Axenov K.V., Moemming C.M., Kehr G., Frohlich R., Erker G. (2010). Structure and dynamic features of an intramolecular frustrated Lewis pair. Eur. J. Chem..

[40] Frynas S., Łastawiecka E., Kozioł A.E., Flis A., Pietrusiewicz K.M. (2019). [4 + 2] Cycloaddition of vinylphosphine oxides to α-oxy-o-xylylene as a route to phosphorylated naphthyl and biaryl scaffolds. J. Org. Chem..

[41] Gawdzik B., Podkościelna B., Bartnicki A. (2006). Synthesis, structure, and properties of new methacrylic derivatives of naphthalene-2,3-diol. J. App. Poly. Sci..

[42] Podkościelna B., Gawdzik B. (2010). Influence of diluent compositions on the porous structure of methacrylate derivatives of aromatic diols and divinylbenzene. App. Surf. Sci..

[43] Podkościelna B. (2011). Synthesis, modification, and porous properties of new glycidyl methacrylate copolymers. J. Appl. Polym. Sci..

[44] Wan Ngah W.S., Hanafiah M.A.K.M. (2008). Adsorption of copper on rubber (Hevea brasiliensis) leaf powder: Kinetic, equilibrium and thermodynamic studies. Biochem. Eng. J..

[45] https://www.dupont.com/content/dam/dupont/amer/us/en/water-solutions/public/documents/en/IER-AmberLite-XAD4-PDS-45-D00781-en.pdf.

[46] Thomas L.C., Chittenden R.A. (1964). Characteristic infrared absorption frequencies of organophosphorus compounds–I. The phosphoryl (P=O) group. Spectrochim. Acta.

[47] Qin P., Wang L.-A., O’Connor J.M., Baldridge K.K., Li Y., Tufekci B., Chen J., Rheingold A.L. (2020). Transition-metal catalysis of triene 6π electrocyclization: The complexation strategy realized. Angew. Chem. Int. Ed..

[48] Podkościelna B. (2011). The highly crosslinked dimethacrylic/divinylbenzene copolymers-characterization and thermal studies. J. Therm. Anal. Calor..

[49] Liu H., Zhu J., Li Q., Li L., Huang Y., Wang Y., Fan G., Zhang L. (2023). Adsorption performance of methylene blue by KOH/FeCl_3_ modified biochar/alginate composite beads derived from agricultural waste. Molecules.

[50] Wiśniewska M., Wawrzkiewicz M., Onyszko M., Medykowska M., Nosal-Wiercińska A., Bogatyrov V. (2021). Carbon-silica composite as adsorbent for removal of hazardous C.I. Basic Yellow 2 and C.I. Basic Blue 3 dyes. Materials.

[51] Majd M.M., Kordzadeh-Kermani V., Ghalandari V., Askari A., Sillanpää M. (2022). Adsorption isotherm models: A comprehensive and systematic review (2010–2020). Sci. Total Environ..

[52] Ayawei N., Ebelegi A.N., Wankasi D. (2017). Modelling and interpretation of adsorption isotherms. J. Chem..

[53] Wawrzkiewicz M., Podkościelna B., Jesionowski T., Klapiszewski Ł. (2022). Functionalized microspheres with co-participated lignin hybrids as a novel sorbents for toxic C.I. Basic Yellow 2 and C.I. Basic Blue 3 dyes removal from textile sewage. Ind. Crops Prod..

[54] Podkościelna B., Wawrzkiewicz M., Goliszek M., Lipke A., Chabros A. (2022). Synthesis and characterization of new polymer sorbents based on EGDMA and cellulose. Physicochem. Probl. Miner. Process..

[55] Öztürk A., Malkoc E. (2014). Adsorptive potential of cationic Basic Yellow 2 (BY2) dye onto natural untreated clay (NUC) from aqueous phase: Mass transfer analysis, kinetic and equilibrium profile. App. Surf. Sci..

[56] Ghorai A., Banerjee S. (2023). Phosphorus-containing aromatic polymers: Synthesis, structure, properties and membrane-based applications. Prog. Polym. Sci..

